# The Precision Resuscitation With Crystalloids in Sepsis (PRECISE) Trial: A Trial Protocol

**DOI:** 10.1001/jamanetworkopen.2024.34197

**Published:** 2024-09-18

**Authors:** Sivasubramanium V. Bhavani, Andre Holder, Danielle Miltz, Rishikesan Kamaleswaran, Sharaf Khan, Kirk Easley, David J. Murphy, Nicole Franks, David W. Wright, Colleen Kraft, Matthew W. Semler, Matthew M. Churpek, Greg S. Martin, Craig M. Coopersmith

**Affiliations:** 1 Department of Medicine, Emory University, Atlanta, Georgia; 2 Emory Critical Care Center, Atlanta, Georgia; 3 Department of Surgery, Duke University, Durham, North Carolina; 4 Rollins School of Public Health, Emory University, Atlanta, Georgia; 5 Department of Emergency Medicine, Emory University, Atlanta, Georgia; 6 Department of Pathology, Emory University, Atlanta, Georgia; 7 Department of Medicine, Vanderbilt University, Nashville, Tennessee; 8 Center for Learning Healthcare, Vanderbilt University, Nashville, Tennessee; 9 Department of Medicine, University of Wisconsin, Madison; 10 Department of Biostatistics and Medical Informatics, University of Wisconsin, Madison; 11 Department of Surgery, Emory University, Atlanta, Georgia

## Abstract

**Importance:**

Intravenous fluids are an essential part of treatment in sepsis, but there remains clinical equipoise on which type of crystalloid fluids to use in sepsis. A previously reported sepsis subphenotype (ie, group D) has demonstrated a substantial mortality benefit from balanced crystalloids compared with normal saline.

**Objective:**

To test the hypothesis that targeting balanced crystalloids to patients with group D sepsis through an electronic health record (EHR) alert will reduce 30-day inpatient mortality.

**Design, Setting, and Participants:**

The Precision Resuscitation With Crystalloids in Sepsis (PRECISE) trial is a parallel-group, multihospital, single-blind, pragmatic randomized clinical trial to be conducted at 6 hospitals in the Emory Healthcare system. Patients with suspicion of group D infection in whom a clinician initiates an order for normal saline in the emergency department (ED) or intensive care unit (ICU) will be randomized to usual care and intervention arms.

**Intervention:**

An EHR alert that appears in the ED and ICUs to nudge clinicians to use balanced crystalloids instead of normal saline.

**Main Outcomes and Measures:**

The primary outcome is 30-day inpatient mortality. Secondary outcomes are ICU admission, in-hospital mortality, receipt of vasoactive drugs, receipt of new kidney replacement therapy, and receipt of mechanical ventilation (vasoactive drugs, kidney replacement therapy, and mechanical ventilation are counted if they occur after randomization and within the 30-day study period). Intention-to-treat analysis will be conducted.

**Discussion:**

The PRECISE trial may be one of the first precision medicine trials of crystalloid fluids in sepsis. Using routine vital signs (temperature, heart rate, respiratory rate, and blood pressure), available even in low-resource settings, a validated machine learning algorithm will prospectively identify and enroll patients with group D sepsis who may have a substantial mortality reduction from used of balanced crystalloids compared with normal saline.

**Results:**

On finalizing participant enrollment and analyzing the data, the study’s findings will be shared with the public through publication in a peer-reviewed journal.

**Conclusions:**

With use of a validated machine learning algorithm, precision resuscitation in sepsis could fundamentally redefine international standards for intravenous fluid resuscitation.

**Trial Registration:**

ClinicalTrials.gov Identifier: NCT06253585

## Key Points

**Question:**

Can an electronic health record (EHR) alert that recommends against use of normal saline in patients with a sepsis subphenotype with potential mortality (group D) benefit from balanced crystalloids result in a lower 30-day mortality rate?

**Findings:**

The Precision Resuscitation With Crystalloids in Sepsis (PRECISE) pragmatic randomized clinical trial intends to test a machine learning algorithm to identify and randomize patients with group D sepsis to usual care or the EHR alert intervention (intention-to-treat). The alert should reduce mortality through decreased normal saline use.

**Meaning:**

The PRECISE trial is one of the first precision trials in fluid resuscitation and could impact sepsis resuscitation guidelines.

## Teaser

This trial protocol of Precision Resuscitation With Crystalloids in Sepsis (PRECISE) intends to test a machine learning algorithm to alert clinicians to the potential benefits of balanced crystalloids compared with normal saline in patients with a group D sepsis subphenotype.

## Introduction

Intravenous fluids are the most common treatment given to hospitalized patients and are an essential part of resuscitation in patients with sepsis.^1,2,3,4^ Despite the ubiquity of intravenous fluids in sepsis, it remains unknown whether it is better to give balanced crystalloids or normal saline, the 2 major classes of crystalloid fluids. More than a dozen randomized clinical trials have enrolled in total more than 35 000 critically ill patients to compare balanced crystalloids vs normal saline, without finding a consistent mortality benefit from one fluid type vs the other.^5,6,7,8,9^ The one-size-fits-all approach has not worked in fluid resuscitation. Taking a precision medicine approach, a machine learning algorithm that uses bedside vital signs to identify a sepsis subgroup (group D) with a 15% absolute reduction in mortality with balanced crystalloids compared with normal saline has been reported.^10^ Compared with patients in the other subgroups, patients with group D sepsis have relatively lower body temperature, heart rate, and respiratory rate, and are hypotensive. In the US, more than 1.5 million patients develop sepsis every year, and almost all patients with sepsis receive fluids.^10,11^ With such a large patient population, even a small reduction in mortality would have a large public health impact.

The Precision Resuscitation With Crystalloids in Sepsis (PRECISE) trial is a parallel-group, multihospital, single-blind, pragmatic randomized clinical trial in which we apply our machine learning algorithm to identify patients with group D sepsis and randomize them to usual care or intervention, with the intervention being an electronic health record (EHR) alert that nudges clinicians to use balanced crystalloids instead of normal saline. We hypothesize that the alert will reduce 30-day inpatient mortality in the intervention cohort through increased use of balanced crystalloids for patients with group D sepsis.

## Methods

**Figure 1.**
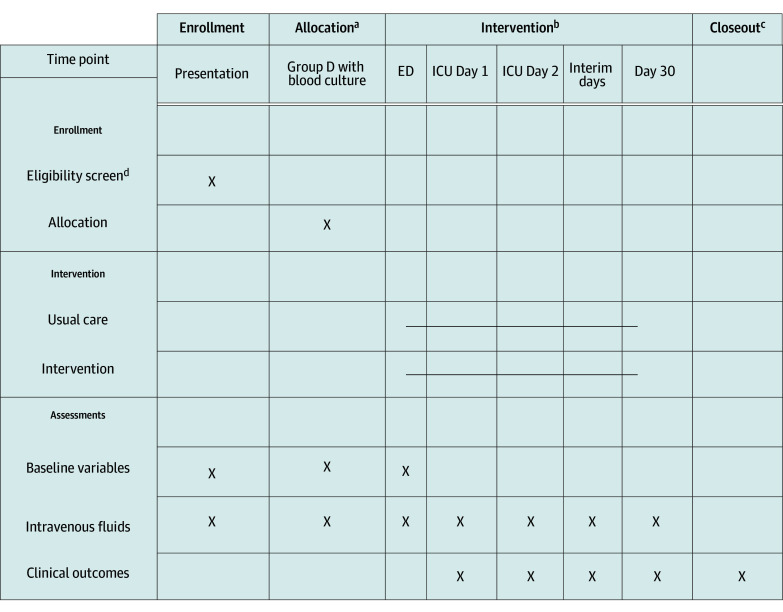
Schedule of Enrollment, Allocation, Intervention, and Study Closeout ED indicates emergency department; ICU, intensive care unit. ^a^Allocation occurs at the point of clinician initiating a normal saline order. ^b^Intervention is only continued in the ED and ICUs. ^c^The study ends after 30 days of hospitalization, discharge from the ICU, or death in the ICU. ^d^Patients aged 18 years or older presenting to study EDs meet eligibility criteria.

This article was prepared in accordance with the Standard Protocol Items: Recommendations for Interventional Trials (SPIRIT) reporting guideline (Figure 1).^12^ The trial is approved by the Emory University Institutional Review Board, with waiver of informed consent given the minimal risk and impracticability of obtaining consent from patients across all emergency departments (EDs), especially in often-emergency situations of fluid resuscitation. Data will not be deidentified. This trial is believed to pose minimal risk because (1) crystalloid resuscitation with either normal saline or balanced crystalloids is part of routine clinical practice, (2) crystalloids in the trial are only administered when the treating clinician orders crystalloids as part of routine clinical practice, and (3) clinicians will have the final decision on what type of crystalloid to administer even in the intervention arm. An independent data and safety monitoring board (DSMB) will monitor the efficacy and safety of the intervention.

### Study Design

The PRECISE trial is a parallel-group, multihospital, single-blind, pragmatic randomized clinical trial being conducted between 2024 and 2025 in 6 EDs and 16 intensive care units (ICUs) across 6 hospitals in the Emory Healthcare system in Atlanta, Georgia. Study hospitals will enroll patients for at least 12 months, with a potentially longer enrollment period based on sample size reestimation on interim analysis.

**Figure 2.**
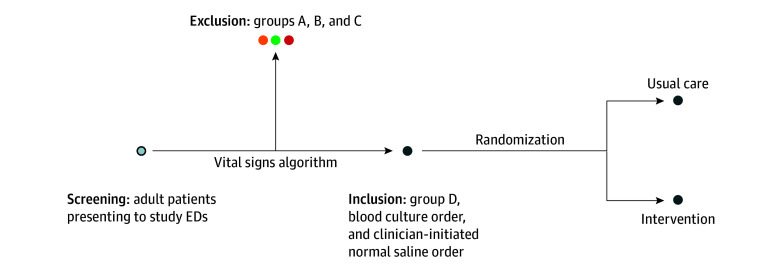
Screening, Inclusion, Exclusion, and Randomization in the Precision Resuscitation With Crystalloids in Sepsis Trial All adult patients presenting to study emergency departments (EDs) will be screened for enrollment. Based on the vital signs trajectory algorithm, patients will be classified as group A, B, C, or D. Those in group D with a blood culture order in whom a clinician initiates a normal saline order are randomized to usual care or intervention.

The study uses a previously validated algorithm that classifies patients into sepsis subphenotypes based on vital signs obtained in the ED.^10,13,14^ Patients who are classified as having group D sepsis, have a blood culture ordered in the ED, and in whom clinicians initiate a normal saline order will be enrolled in the trial and randomized to usual care vs intervention to compare the primary outcome of 30-day inpatient mortality (Figure 2). The intervention is an EHR alert that nudges clinicians toward ordering balanced crystalloids rather than normal saline, as preliminary data suggest that balanced crystalloids confer a substantial mortality benefit in patients with group D sepsis.

### Algorithm Description

The vital signs trajectory algorithm uses vital signs (temperature, heart rate, respiratory rate, and blood pressure) from hospital presentation to classify patients into 1 of 4 subphenotypes (group A, B, C, and D). The algorithm was developed by applying group-based trajectory modeling to vital signs from the first 8 hours of hospital presentation in a cohort of 12 413 patients with suspicion of infection admitted to 4 Emory Healthcare hospitals between January 2014 and December 2017. The algorithm has been validated in a cohort of 8256 patients hospitalized with suspicion of infection between 2018 and 2019 and in 7065 patients hospitalized with COVID-19 between 2020 and 2022. The model was originally developed as a trajectory algorithm, with each subphenotype defined by a set of 5 unique polynomial functions, and with each function describing a vital sign as a function of time from presentation to the hospital (eg, heart rate = β_0_ + β_1_ × time + β_2_ × time^2^). The model has also been tested as an intercept algorithm, using only the first set of vital signs, such that each subphenotype is defined by a set of 5 unique intercepts (eg, heart rate = β_0_). In a secondary analysis of the Isotonic Solutions and Major Adverse Renal Events Trial, there was 80% agreement in classification between the parsimonious intercept model and the criterion standard model, with 90% agreement in specifically group D classification.^6,10^ For the PRECISE trial, the intercept model is being used to classify patients rapidly in the ED with a single set of vital signs, rather than awaiting a full 8 hours of vital signs documentation. Since crystalloid fluid resuscitation starts soon after hospital presentation, the earlier the patient is classified, enrolled, and randomized, the higher the likelihood of observing a benefit from the intervention.

### Population

**Figure 3.**
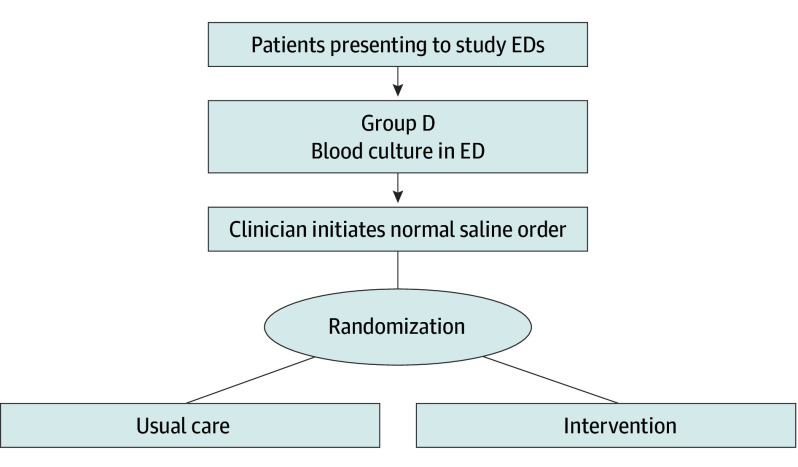
Enrollment and Randomization in the Precision Resuscitation With Crystalloids in Sepsis Trial Broad inclusion criteria of patients presenting to study emergency departments (EDs) who are classified as group D and have a blood culture ordered. If a clinician initiates a normal saline order, the patients are randomized to usual care vs intervention arms.

All adults (aged ≥18 years) presenting to study EDs will be screened. Inclusion criteria are (1) blood culture order in the ED, (2) classification as group D by the vital signs trajectory algorithm, and (3) clinician-initiated normal saline order. Patients who meet the inclusion criteria will be randomized to intervention vs usual care. If study patients are discharged and readmitted to a study hospital during the study period, they will be eligible for reenrollment (Figure 3).

### Randomization and Allocation

Patients will be randomized to usual care vs intervention using software within the EHR in a simple 1:1 ratio without stratification. Randomization occurs at the point of clinicians initiating a normal saline order.

For patients randomized to the intervention arm, the EHR alert will fire in the ED and the ICU every time normal saline is ordered until the first of the following events: ICU discharge, death, or 30 days of hospitalization. The study intervention will not be continued in the wards. For patients randomized to the usual care arm, there will be no EHR alert in response to fluid orders.

### Protocol Update

This protocol document was updated on July 4, 2024, and the trial eligibility criteria were modified to match the randomization process as operationalized within the EHR. The original eligibility criteria were patients with group D sepsis with a blood culture ordered in the ED. However, the EHR randomization process occurs only at the point of the clinician initiating a normal saline order. Thus, the updated trial eligibility criteria are patients with group D sepsis with a blood culture order in the ED for whom clinicians initiate an order for normal saline.

### Concealment and Blinding

The PRECISE study is single-blind (ie, patients are blinded to the allocation). In the usual care arm, the clinicians will not receive any feedback from the algorithm and will not know the group classification of their patient. In the intervention arm, if a clinician orders normal saline in a patient classified as having group D sepsis, there will be an EHR alert and the clinician will necessarily be unblinded to the allocation.

### EHR Alert

The EHR alert will fire when a clinician orders normal saline in a patient with group D sepsis who is randomized to the intervention arm. The alert will announce that there is evidence for a reduced mortality risk from use of balanced crystalloids in this patient and will ask the clinician to choose either lactated Ringers or PlasmaLyte (Baxter), unless there is a clinical indication to continue normal saline. The alert will fire on normal saline orders for maintenance infusions and boluses, but not on normal saline ordered as part of medication diluents and carrier fluid for intravenous medications.

### Outcomes

The primary outcome is 30-day inpatient mortality. Secondary outcomes include ICU admission, in-hospital mortality, receipt of vasoactive drugs, receipt of new kidney replacement therapy, and receipt of mechanical ventilation (vasoactive drugs, kidney replacement therapy, and mechanical ventilation are counted if they occur after randomization and within the 30-day study period). Process outcomes include volume of normal saline and percentage of alerts accepted.

### Statistical Analysis

The primary outcome is 30-day inpatient mortality compared between the usual care and intervention arms using intention-to-treat analysis (ie, all patients randomized to intervention vs usual care regardless of whether the intervention resulted in a change in fluid choice). The outcome of 30-day inpatient mortality is measured for the index hospitalization. Readmissions and subsequent deaths are not counted toward the primary outcome for the initial hospitalization, but are considered for the readmission hospitalization. The primary analysis will be an assessment for a difference in 30-day inpatient mortality between the arms with a 2-sided *z* test with continuity correction and pooled variance. Thirty-day inpatient mortality rates will be determined using 95% CIs within each study arm and for the observed difference between study arms.

The secondary analysis for primary and secondary outcomes will be a generalized mixed-effects linear model (ie, log-binomial regression model) to estimate the outcome, adjusted for baseline characteristics including age, sex, race and ethnicity, and Sequential Organ Failure Assessment (SOFA) score at randomization. Race and ethnicity will be included in the analyses given potential disparities in treatments and health outcomes by race. The model will also be adjusted for month of the trial, with an interaction term between month and treatment allocation, to evaluate for temporal trends in the efficacy of the intervention (eg, increasing use of balanced crystalloids in usual care could result in decreased separation of the study arms). The hospital to which the patient is admitted will be defined as a random effect (random intercept) in the mixed model. In a separate sensitivity analysis, the hospital to which the patient is admitted will be defined as a fixed effect to evaluate the robustness of the findings. The covariate-adjusted 30-day inpatient mortality rates will be determined using 95% CIs for each study cohort and for the observed difference between the usual care and intervention arms in 30-day inpatient mortality rates. Relative risks will be calculated to measure the degree of association between individual risk factors and 30-day inpatient mortality. The SAS GLIMMIX Procedure, version 9.4 (SAS Institute Inc) will be used to implement the log-binomial regression model.

The process outcome of volume of normal saline administered per patient will be compared in unadjusted analysis between the intervention and usual care arms. The total number of alerts generated and the percentage of alerts accepted in the intervention arm will be reported.

Secondary analysis of the primary outcome will be performed on the subset of randomized patients who were admitted to the ICU and thus would have potential exposure to intervention in both the ED and ICU settings. Any additional outcomes or analyses will be clearly identified as post hoc and will be considered hypothesis-generating.

### Subgroup Analyses for 30-Day Inpatient Mortality

The heterogeneity of intervention effects across levels of a baseline variable will be investigated using a statistical test for interaction.^15^ The effect of intervention on 30-day inpatient mortality will be evaluated by the following prespecified subgroups: age, sex, severity of illness (quartiles of SOFA), and kidney injury on hospital presentation (KDIGO stage <2 [less severe] vs KDIGO stage ≥2 at enrollment). Effect of intervention in subgroups will be determined by including the interaction between the intervention and subgroup in the log-binomial regression model. Sensitivity analysis will be performed on patients with presence of sepsis defined by *International Statistical Classification of Diseases and Related Health Problems, 10th Revision* coding on discharge.

### Safety End Points

The patient population in the ED and ICU will experience many physiologic abnormalities due to the severity of their illness and the standard treatments received. Both normal saline and balanced crystalloids are standard treatments, and the final treatment decision between the 2 crystalloid fluids is left to the treating clinician. The adverse events are evaluated within the framework of the primary and secondary outcomes described. Any unexpected treatment-related serious adverse events will be identified and reported.

### Sample Size and Power Considerations

The power analysis was performed using Emory Healthcare system retrospective data, with mortality estimates based on proportion of normal saline vs balanced crystalloids received. Usual care has an expected mortality rate of 6%, and intervention has an expected mortality rate of 3%. To detect a proportion difference (P2 – P1) of 0.03 (or P1 of 0.03) with 90% power, the number of participants needed will be 1001 in the intervention arm and 1001 in the usual care arm (Table 1). The power was computed using the tests for 2 proportions module from PASS 2023, version 23.0.1 (NCSS LLC).

**Table 1.** Power and Sample Size for Hypothesis Tests of the 30-Day Inpatient Mortality Difference Between Usual Care and Intervention^a^

Test	Sample size assumptions and results					
	Usual care P2	Intervention P1	Difference (P2 − P1)	No. of participants		Statistical power, %
				Per group	Total	
1	0.06	0.03	0.03	748	1496	80
2	0.06	0.04	0.02	1863	3726	80
3	0.06	0.03	0.03	1001	2002	90
4	0.06	0.04	0.02	2493	4986	90

^a^ The usual care group mortality rate (P2) is assumed to be 0.06. To detect a proportion difference (P2 − P1) of 0.03 (or P1 of 0.03) with 90% power, the number of participants needed will be 1001 in the intervention arm and 1001 in the usual care arm.

### Interim Analysis

The DSMB will conduct 2 interim analyses. A focus for the interim analyses is the effectiveness of the intervention in changing clinician ordering behavior. To decrease the use of normal saline in the intervention arm, an education program for ED and ICU clinicians has occurred before trial enrollment. On both the 3- and 6-month interim analyses, if the rate of normal saline use is greater than 25%, an additional targeted education campaign will be undertaken. At both interim analyses, the DMSB will monitor for meeting a stopping boundary for efficacy, with an unadjusted primary outcome (30-day mortality) difference between groups using a χ^2^ test with a significance level of *P* < .001. The conservative Haybittle-Peto boundary is used so that the final analysis can be performed with an unchanged significance level (2-sided *P* < .05). At the 3-month interim analysis, the DSMB can suggest a sample size reestimation if the mortality rate in the usual care arm differs clinically (eg, 9% vs 6%) from the estimated 6% mortality rate. The DSMB can also suggest a sample size reestimation if the baseline prevalence of normal saline use is significantly lower than the estimated 60%.

No futility boundary to mortality difference will be applied given that even a small clinical benefit from the intervention could have a large impact given the ubiquity of crystalloid fluid therapy. In addition to prescheduled data reviews and planned safety monitoring, the DSMB may be called on for ad hoc reviews. The DSMB will review any event that potentially impacts safety at the request of the DSMB chair. The DSMB will comprise an academic intensivist from a separate university, an academic intensivist from a nonparticipating hospital in Atlanta, and an independent biostatistician.

### Data Management

Given the EHR-embedded nature of this pragmatic trial, data collection will occur through EHR data extraction, and all data will be routinely collected information, including demographic characteristics, vital signs, laboratory values, medication orders, and vital sign status at hospital discharge. At the end of every study month, data will be extracted for all patients that month who were randomized. Baseline variables, liters of crystalloid fluids administered, and outcomes will be collected on all randomized patients. After study completion, there will be an additional data extraction completed at 1 month following study completion to finalize patient data. If any patient remains hospitalized at the end of the 1-month period following study completion, the patient’s in-hospital mortality status will be documented as incomplete. No other instances of missing outcomes data or loss to follow-up are expected, as all data will be extracted from the EHR. Monthly hospital-level aggregate reports will be sent to the DSMB by allocation group to allow evaluation of crystalloid fluid distribution, without information on outcomes. At the interim analyses, a detailed patient-level report will be sent to the DSMB unblinded to allocation with information on outcomes (Table 2).

**Table 2.** Template for Patient-Level Data for Enrolled Patients in the Precision Resuscitation With Crystalloids in Sepsis (PRECISE) Trial

Pt	Admission date	Randomization	ICU^a^	Baseline variables^b^	NS^c^	BC^c^	30-d mortality	In-hospital mortality^d^
1	MM/DD/YY	Intervention	No	Variable	NS value	BC volume	Rate	Rate
2	MM/DD/YY	Intervention	Yes	Variable	NS value	BC volume	Rate	Rate
3	MM/DD/YY	Usual care	Yes	Variable	NS value	BC volume	Rate	Rate
4	MM/DD/YY	Intervention	Yes	Variable	NS value	BC volume	Rate	Rate
5	MM/DD/YY	Usual care	Yes	Variable	NS value	BC volume	Rate	Rate
6	MM/DD/YY	Usual care	No	Variable	NS value	BC volume	Rate	Rate
7	MM/DD/YY	Usual care	No	Variable	NS value	BC volume	Rate	Rate
8	MM/DD/YY	Intervention	Yes	Variable	NS value	BC volume	Rate	Rate

Abbreviations: BC, balanced crystalloids; ICU, intensive care unit; MM/DD/YY, month, day, year; NS, normal saline; Pt, patient identifier.

^a^ If patient was admitted from the emergency department to an ICU.

^b^ There will be individual columns, including baseline variables such as age, sex, race and ethnicity, hospital, and Sequential Organ Failure Assessment score.

^c^ Liters of crystalloid fluids administered during the 30-day study period.

^d^ In-hospital mortality will be marked as incomplete if the patient remains in the hospital 1 month after study completion.

### Dissemination of Trial Findings

On finalizing participant enrollment and analyzing the data, we will share the study’s findings with the public by publishing a peer-reviewed article and entering the results into the ClinicalTrials.gov database. We will ensure that the entire study protocol and the statistical code are accessible to the public. The determination of authorship will adhere to the standards set by the International Committee of Medical Journal Editors.

### Data Sharing Plan

On publication, deidentified patient data will be made available to researchers who provide a methodologically sound proposal, subject to approval by the study team. Requests for data sharing should be directed to the corresponding author, and data will be accessible for at least 5 years following the publication of the primary results.

## Discussion

The ultimate impact of the PRECISE trial is use of the most commonly available data (vital signs) to guide the most commonly administered therapy (intravenous fluids) in one of the most common causes of preventable mortality (sepsis) responsible for 1 in 5 deaths worldwide.^16^ Using routine vital signs available even in low-resource settings, we aim to prospectively identify a sepsis subgroup with a significant mortality reduction from use of balanced crystalloids compared with normal saline. Precision resuscitation in sepsis could fundamentally redefine international standards for intravenous fluid resuscitation. Given the more than 48 million annual cases of sepsis worldwide, the precision resuscitation approach could dramatically reduce mortality from sepsis.

The primary research question is whether an EHR alert that recommends use of balanced crystalloids to a sepsis subphenotype (previously shown to have mortality benefit from balanced crystalloids^10^) will result in a lower 30-day mortality rate. Given the complexity of the study question and design, there will be numerous generalizable contributions from this trial. First, the trial will provide generalizable knowledge on fluid management in the group D subphenotype. If the significant mortality benefit from balanced crystalloids in group D is validated, the PRECISE trial could inform international guidelines, such as the Surviving Sepsis Guidelines, and could change the standard of care for fluid resuscitation in sepsis.^1^ Second, the trial may demonstrate the feasibility and limitations of real-time subphenotyping for precision medicine trials in sepsis. While there has been over a decade of research on sepsis subphenotypes, this will be one of the first clinical trials to use subphenotypes for precision enrollment.^17,18,19,20,21^ Third, the trial will allow us to understand the human factors in implementation of EHR-based interventions. We are aiming to decrease normal saline use in the intervention arm through the EHR alert design and a concerted education campaign. The resulting rate of normal saline use will be important in understanding the potential ceiling to behavioral changes through EHR alerts.

### Limitations

There are several potential limitations to the validity and generalizability of the clinical trial results. First, the influence of the intervention on patient outcomes depends on the effect of the intervention on changing clinician behavior. If the intervention and education campaign fail to shift clinician-ordering behavior, there may be limited separation in crystalloid ordering patterns between usual care and intervention. Second, the influence of the intervention depends on baseline fluid prescription practices, which may change over time. Third, the study does not encompass nonclinician-ordered crystalloid fluids, such as some fluids given in emergency situations (eg, bedside nurse grabs a fluid bag to administer in an acutely decompensating patient), carrier or piggyback fluids given with intravenous medications, fluid given as flushes, or fluids used for medication dilution. This approach, while not comprehensive for all fluid administration, will likely reflect generalizability to outside health care systems. Fourth, the study uses the intercept model, which may not accurately identify all patients with group D sepsis compared with the criterion standard 8-hour vital signs trajectory model.

## Conclusions

To date, the PRECISE trial represents one of the first precision enrollment trials in sepsis and fluid resuscitation, and the findings could markedly impact sepsis resuscitation guidelines.

## Notes

Supplement.
Data Sharing Statement
