# Supplementary material for: The Precision Resuscitation With Crystalloids in Sepsis (PRECISE) Trial: A Trial Protocol
Source: JAMA Netw Open. 2024 Sep 18;7(9):e2434197. doi: 10.1001/jamanetworkopen.2024.34197 (PMC11411385; doi:10.1001/jamanetworkopen.2024.34197)
Supplement: Supplement. — Data Sharing Statement [file jamanetwopen-e2434197-s001.pdf]

# Data Sharing Statement

Bhavani. The Precision Resuscitation With Crystalloids in Sepsis (PRECISE) Trial. *JAMA Netw Open*. Published September 18, 2024. doi:10.1001/jamanetworkopen.2024.34197

**Data**
**Data available:** Yes
**Data types:** Deidentified participant data
**How to access data:** Requests for data sharing should be directed to the corresponding author (sbhava2@emory.edu), and data will be accessible for a period of at least five years following the publication of the primary results.
**When available:** With publication

**Supporting Documents**
**Document types:** None

**Additional Information**
**Who can access the data:** De-identified patient data will be made available to researchers who provide a methodologically sound proposal, subject to approval by the study team.
**Types of analyses:** Any secondary analyses of the trial results.
**Mechanisms of data availability:** After approval of a proposal and a signed data access agreement.
